# Cumulative Dose Analysis in Adaptive Carbon Ion Radiotherapy for Locally Advanced Non-Small Cell Lung Cancer

**DOI:** 10.3390/cancers17162709

**Published:** 2025-08-20

**Authors:** Zhuojun Ju, Makoto Sakai, Xiangdi Meng, Nobuteru Kubo, Hidemasa Kawamura, Tatsuya Ohno

**Affiliations:** 1 Department of Radiation Oncology, Gunma University Graduate School of Medicine, Maebashi 3718511, Japan; m2320017@gunma-u.ac.jp (Z.J.); m2320043@gunma-u.ac.jp (X.M.); tohno@gunma-u.ac.jp (T.O.); 2 Gunma University Heavy Ion Medical Center, Maebashi 3718511, Japan; kubo@gunma-u.ac.jp (N.K.); kawa@gunma-u.ac.jp (H.K.)

**Keywords:** carbon ion radiotherapy, adaptive radiotherapy, locally advanced non-small cell lung cancer, cumulative dose distribution, weekly computed tomography

## Abstract

**Objectives**: This study aimed to assess the precision of dose delivery to the target in adaptive carbon ion radiotherapy (CIRT) for locally advanced non-small cell lung cancer (LA-NSCLC) in cumulative dosimetry. **Methods:** Forty-six patients who received CIRT were included (64 Gy[relative biological effectiveness, RBE] in 16 fractions) with treatment plan computed tomography (CT) and weekly CT scans. Offline adaptive radiotherapy (ART) was administered if the dose distribution significantly worsened. Daily doses were calculated from weekly CTs and integrated into plan CT scans using deformable image registration. The dosimetry parameters were compared between the as-scheduled plan and adaptive replan in patients receiving ART. Survival outcomes and toxicity were compared between the ART and non-ART groups. **Results:** ART was implemented for 27 patients in whom adaptive replans significantly increased the median V_98%_ of the clinical tumor volume from 96.5% to 98.1% and D_98%_ from 60.5 to 62.7 Gy(RBE) compared with the as-scheduled plans (*p* < 0.001). The conformity and uniformity of the dose distribution improved (*p* < 0.001), with no significant differences in the doses to normal tissues (lungs, heart, esophagus, and spinal cord) from the as-scheduled plans (*p* > 0.05). The ART and non-ART groups demonstrated comparable local control, progression-free survival, and overall survival (*p* > 0.05). No grade 3 or higher radiation-related toxicities were observed. **Conclusions:** ART enhanced target dose coverage while maintaining acceptable normal tissue exposure, supporting weekly CT monitoring integration during CIRT for the timely intervention for anatomical variations, ensuring precise dose delivery in LA-NSCLC.

## Simple Summary

Carbon ion radiotherapy (CIRT) takes advantage of the Bragg peak to achieve highly localized energy deposition within the tumor while minimizing exposure to surrounding normal tissues. However, its benefits may be compromised by anatomical changes during treatment, necessitating adaptive strategies to maintain dosimetric precision. This study aimed to assess the accuracy of dose delivery in adaptive radiotherapy (ART) for locally advanced non-small cell lung cancer, focusing on cumulative dose evaluation. Weekly CT scans were performed in 46 patients, and 27 were found to require ART. Compared with the as-scheduled plan, their ART plan significantly improved tumor coverage and conformity, without increasing exposure to normal tissues. Moreover, the ART group showed no decrease in survival or increase in toxicity. These findings support the use of weekly CT and adaptive planning to ensure effective treatment and provide a foundation for future research into the clinical benefits of ART in CIRT.

## 1. Introduction

In carbon ion radiotherapy (CIRT), the carbon ion beam delivers a large part of its energy within the intended targeted tumor, creating a distinct Bragg peak and thereby minimizing damage to the surrounding and traversed normal tissues [1,2,3]. Compared with photon radiotherapy (RT), CIRT offers a higher relative biological effectiveness (RBE), approximately three times greater in the tumor area [3,4]. Furthermore, larger tumors often develop radioresistance because of hypoxia; however, the lower dependence of CIRT on oxygen allows for enhanced therapeutic efficacy under such conditions [5]. Despite these advantages, the high sensitivity of CIRT to anatomical changes limits its therapeutic potential [6], particularly in the thoracic region, necessitating adaptive replanning [7,8].

Adaptive RT (ART) is a technique that dynamically adjusts treatment plans through repeated simulation, planning, and quality assurance to address dose distribution deterioration caused by anatomical deviations [7,9,10,11]. The treatment team typically relies on daily or weekly computed tomography (CT) to identify unacceptable anatomical variations and then makes adaptive decisions accordingly [12]. Evidence increasingly supports that ART can enhance dosimetry and clinical outcomes, allowing for smaller safety margins [10,11].

Lung cancer presents a high demand for ART in CIRT, driven by its high incidence and significant tumor movability [13]. Non-small cell lung cancer (NSCLC) accounts for approximately 85% of all lung cancer cases, with >30% of patients diagnosed at a locally advanced stage (LA-NSCLC), indicating that many patients have large tumor volumes or lymph node metastases [14,15]. Despite advancements in tumor motion management techniques, such as gated CT and four-dimensional CT [16], these techniques mainly address intrafractional variations and are insufficient to address suboptimal dose distributions due to interfractional anatomical variations, potentially compromising treatment efficacy. Consequently, timely cumulative dose assessment and adaptive treatment planning are crucial in clinical practice.

However, ART is insufficiently implemented in CIRT. CIRT differs from photon and proton therapies in several ways, including a shorter treatment period and a steep penumbra. Although the study has examined cumulative dosing in CIRT for stage I lung cancer [8,17], locally advanced lung cancer involves greater anatomical variability, making cumulative dose assessment and ART implementation even more important. Jia et al. evaluated the recovering dose of CIRT-ART in target coverage for LA-NSCLC on a single treatment day [7]; however, their study did not include accumulated dose calculations and did not assess survival or toxicity outcomes. To evaluate the total dose distribution and its outcomes, periodic CT scans and support from deformable image registration (DIR) technology are required [9,13,14].

This study aimed to assess the potential dosimetry benefits of ART in CIRT for LA-NSCLC by calculating the cumulative dose distribution and comparing survival outcomes and adverse events between the ART and non-ART groups.

## 2. Materials and Methods

### 2.1. Patients

Forty-six patients with LA-NSCLC (stage IIB–IIIC, American Joint Committee on Cancer 8th Edition), confirmed by histopathology or clinical imaging following internal discussion, were consecutively enrolled in this study. All patients received passive scattering CIRT either owing to meeting radiotherapy indications, medical inoperability, or personal refusal of surgery from October 2018 to September 2023. None of the patients had a history of prior thoracic radiotherapy or systemic treatment. The clinical characteristics of the patients are summarized in Table 1. This retrospective study was approved by the Institutional Review Board of Gunma University (No. HS2024-164).

### 2.2. Image Acquisition

Planning CT was performed 1–2 weeks before CIRT to create a treatment plan (original plan). Depending on tumor location, the patients were positioned either supine or prone in a body cradle, which could be rolled ±15° around the superior–inferior axis to align the target with either the horizontal or vertical fixed irradiation ports [17,18]. A customized thermoplastic shell (Shellfitter; Sanyo Polymer Industrial, Nara, Japan) and water-hardened polymer pillow (Moldcare; ALCARE, Tokyo, Japan) were used to immobilize the patients [17,18]. For all patients in the cohort, CT images were captured at 30% of the respiratory wave amplitude near the exhalation peak using 4D CT and respiratory gating technology (monitored by a respiration laser sensor; AZ-733V, Anzai Medical, Tokyo, Japan) [17], serving as the plan CT images. The same settings and parameters were applied in subsequent CT scans and during treatment. To ensure the reproducibility of treatment planning and tumor position, CT scans were repeated using the same technique 1–2 days before CIRT (confirm CT) and periodically during CIRT (weekly CT), using the in-room fan-beam CT. The layout and imaging parameters of the in-room CT system were identical to those used in the CT simulation room, and the patient’s positioning and immobilization method during image acquisition were exactly the same as those used at simulation and during treatment [17,18]. Before each fraction, all necessary treatment parameters were carefully checked and confirmed by the treatment staff. To reduce setup and angular errors, daily orthogonal X-ray image guidance with six-degrees-of-freedom bone matching was performed, maintaining translational and rotational residual errors within acceptable limits, and any deviations beyond tolerance were corrected on the treatment couch [17,18,19].

### 2.3. Treatment Planning

On plan CT, the radiation oncologist first delineated the gross tumor volume (GTV) and then expanded it outward by 5 mm to create the clinical target volume (CTV). Respiratory gating technology was used to determine the tumor position within the gating window, allowing for the calculation of tumor motion in all directions (superior, inferior, left, right, anterior, and posterior). The internal margin (IM) was defined as one-third of the extent of tumor motion in each direction [16]. The planning target volume was created by anisotropically adding the square root of the sum of the squares of the IM and 3 mm setup margin to the CTV.

The pencil beam algorithm in the XiO-N treatment planning system (Elekta, Stockholm, Sweden; Mitsubishi Electric, Tokyo, Japan) was used at our facility, with the clinical dose calculated based on the physical dose and RBE using the HIMAC model [20,21,22], expressed as “Gy(RBE).” For the LA-NSCLC treatment plan, the total dose was 64 Gy(RBE) administered in 16 fractions, four fractions per week. Four fields (two vertical and two horizontal) were used to minimize damage to surrounding normal tissues, and one field was delivered per treatment day.

### 2.4. Planning Assessment and Adaptive Replanning

Confirm and weekly CT scans were performed to evaluate the quality and reproducibility of the treatment plan. Using MIM Maestro (version 7.3.2; MIM Software Inc., Cleveland, OH, USA), the plan CT and confirm CT (or weekly CT) were fused and matched according to the positioning method of the treatment. To ensure consistency in data acquisition and reduce potential errors for analysis, bone matching was uniformly applied for all patients. After image matching, confirm CT or weekly CT with regions of interest was transferred to the XiO-N system, and the dose distribution was calculated using the same parameters as the original treatment plan. Adaptive replanning was triggered if 95% of the prescribed dose failed to cover the CTV or if the irradiation dose to normal tissues exceeded acceptable levels (Table A1). After an internal discussion, the tumor target-related contour and/or original treatment plan was optimized to ensure adequate target area dosing while reducing normal tissue exposure to the same low level as in the original plan. Subsequent CIRT fractions for the patient adhered to the revised plan following adaptive replanning.

**Figure 1:**
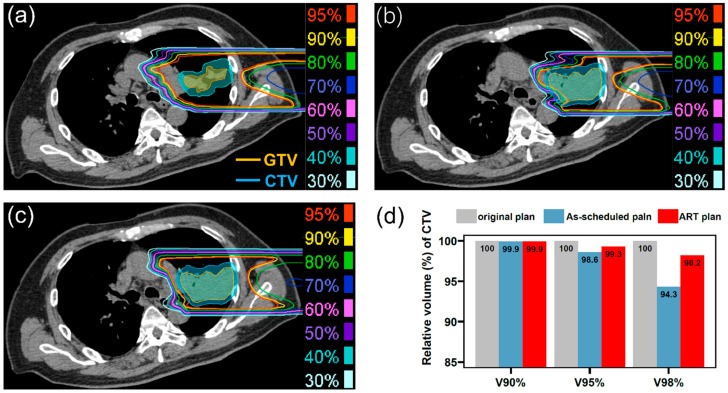
(**a**) Dose distribution of a field in the original treatment plan on the plan CT (4 Gy[RBE]), (**b**) dose distribution of the field in the as-scheduled plan on the weekly CT (4 Gy[RBE]), (**c**) dose distribution of adapted field on the weekly CT (4 Gy[RBE]), and (**d**) dose–volume histogram parameters between these plans. GTV, gross tumor volume; CTV, clinical target volume; ART, adaptive radiotherapy; RBE, relative biological effectiveness.

Figure 1 illustrates an example of the ART process. The original treatment plan is presented in Figure 1a. In the third weekly CT scan (after 13 fractionated irradiations), the tumor volume had increased, and the as-scheduled plan could no longer provide adequate target coverage (Figure 1b). After an internal discussion, we decided to adjust the beam field to ensure effective dose coverage of the tumor (Figure 1c). After adaptive replanning, CTV coverage with 98% of the prescribed dose in this direction increased from 94.3% to 98.2% (Figure 1d).

### 2.5. Accumulated Dose Calculation

**Figure A1:**
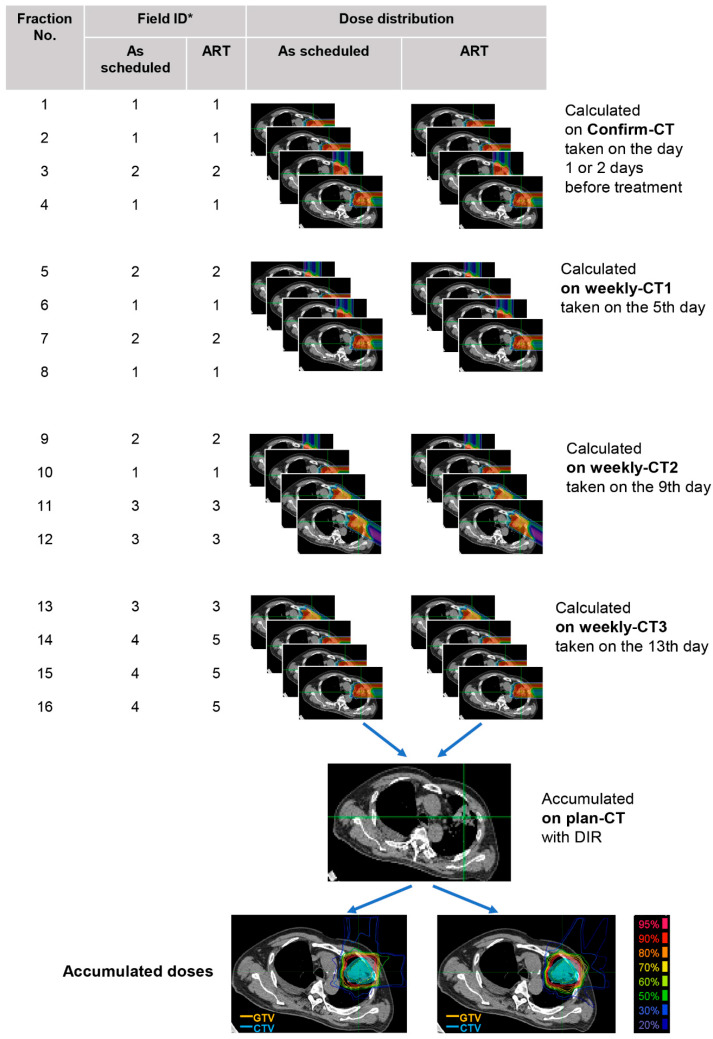
Cumulative dose calculation flowchart. *: Field ID represents the treatment irradiation field number (default 1–4). During CIRT, new fields are created when dose deterioration was detected by adjusting the original ones—as shown by Field 5 in this example, which was re-optimized from Field 4. Abbreviations: ART, adaptive radiotherapy; DIR, deformable image registration; CTV, clinical target volume; GTV, gross tumor volume.

Cumulative dose distributions were calculated by integrating multiple CT scans, as shown in Figure A1. Each patient underwent at least four CT scans (one confirm CT and three weekly CTs) at the facility. The cumulative dose for the irradiation fields used during the first week was calculated using the confirm CT. For the second week fields, the first weekly CT was used. Subsequently, all irradiation fields from the four CTs were integrated into the plan CT using DIR from the MIM. DIR deforms and aligns the anatomical structures and irradiation fields from each CT scan to register the doses from the confirm CT and weekly CTs onto the plan CT coordinate system to generate the cumulative dose distribution. In this study, the “as-scheduled plan” was generated on the plan CT by integrating the initial irradiation fields, while the “adaptive plan” was created by integrating the actual irradiation fields.

The dose–volume histogram parameters evaluated in this study included the volume percentage of the CTV covered by 90% (V_90%_), 95% (V_95%_), and 98% (V_98%_) of the prescription dose and doses covering 90% (D_90%_), 95% (D_95%_), and 98% (D_98%_) of the CTV. For normal tissues, this study evaluated V_5 Gy(RBE)_, V_10 Gy(RBE)_, V_20 Gy(RBE)_, and the mean dose for the total and ipsilateral lungs; V_40 Gy(RBE)_ and the mean dose for the heart; and the mean and maximum doses for the esophagus and maximum doses for the spinal cord.

The conformity index (CI) and homogeneity index (HI) were calculated to assess the precision and uniformity of the irradiation dose delivered to the CTV [7,23]. The closer the HI and CI values were to 1, the more uniform the dose distribution and the better the conformity. The formula is as follows:

$$HI=\frac{D_{CTV,5\%}}{D_{CTV,95\%}},\;CI=\frac{V_{CTV,98\%}^{2}}{V_{CTV}\times V_{98\%}}$$

where *D*_*CTV*,5%_ and *D*_*CTV*,95%_ are the doses received by 5% and 95% of the CTV, respectively, and *V*_*CTV*,98%_ is the volume of the CTV that received 98% of the prescribed dose. *V_CTV_* is the volume of the CTV, and *V*_98%_ is the volume inside the isodose curve for 98% of the prescribed dose.

### 2.6. Follow-Up and Survival Outcomes

Radiation-related toxicity was assessed according to the Common Terminology Criteria for Adverse Events version 4.0, with weekly evaluations conducted during RT. For the first 6 months after CIRT, the patients underwent monthly physical examinations, toxicity assessments, and chest examinations, with chest CT scans conducted every 3 months. After this period, the follow-up schedule was modified every 6 months and continued for 5 years. During these visits, an annual 18F-fluorodeoxyglucose positron emission tomography scan was performed. Additional tests were conducted if recurrence or metastasis was suspected.

The primary survival outcomes were local control (LC), progression-free survival (PFS), and overall survival (OS). Time intervals for recurrence, metastasis, or death were calculated from the end of CIRT to the occurrence of an event or the date of the last follow-up.

### 2.7. Statistical Analyses

Continuous and categorical variables were analyzed using Mann–Whitney U and chi-squared tests, respectively. For intragroup comparisons, the paired Mann–Whitney U test was used. LC, PFS, and OS were estimated using the Kaplan–Meier method, and survival outcomes between the ART and non-ART groups were compared using the log-rank test. A *p*-value of <0.05 was considered statistically significant. All statistical analyses were performed using R (version 4.4.0).

## 3. Results

### 3.1. Patient Characteristics

Forty-six patients were included in this study, with a mean age of 73.6 (±9.5 years). Among them, 58.7% (n = 27) of the patients underwent adaptive replanning. Compared with the non-ART group, the patients in the ART group had larger tumor volumes (42.5 mL [interquartile range (IQR): 21.2, 90.4 mL] vs. 25.6 mL [2.7, 45.3 mL], *p* = 0.037). No significant differences were observed between the two groups in terms of the other characteristics (*p* > 0.05) (Table 1).

### 3.2. Characteristics of Adaptive Radiotherapy

Thirty-eight adaptive responses were triggered in 27 patients owing to unsatisfactory dose distributions (n = 13), uncomfortable lying position (n = 1), increased (n = 9) or decreased (n = 3) tumor volume, and overdose to normal tissues (n = 14; four esophagus, five bronchus, four spinal cord, and one lung). Among them, two patients required adaptive replanning due to tumor enlargement detected on the confirm CT scan the day before CIRT. Additionally, seven patients required replanning twice, and two patients required replanning three times. Overall, the median fraction in which ART was triggered was 7 (range, 1–14), with 11 (40.7%) cases occurring in the first week of treatment (confirm CT), followed by the second week of treatment (first weekly CT) (n = 7, 25.9%), third week of treatment (second weekly CT) (n = 6, 22.2%), and fourth week of treatment (third weekly CT) (n = 3, 11.1%).

### 3.3. Comparison of Target and Normal Tissue Coverage Between As-Scheduled Plan and Adaptive Replan

**Figure 2:**
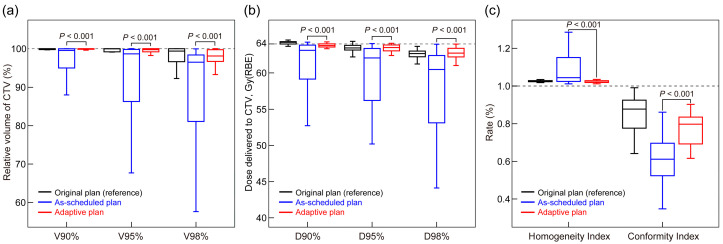
Comparison of CTV cumulative dose distributions between as-scheduled plan and adaptive replan: (**a**) CTV coverage receiving 90% (V_90%_), 95% (V_95%_), and 98% (V_98%_) of the prescribed dose; (**b**) dose delivering to 90% (D_90%_), 95% (D_95%_), and 98% (D_98%_) of CTV; and (**c**) homogeneity and conformity indices. *p*-values were calculated using the paired Mann–Whitney U test. CTV, clinical target volume; RBE, relative biological effectiveness.

A comparison between the as-scheduled plan and adaptive replan for 27 patients receiving ART revealed that adaptive replanning significantly improved the CTV coverage. The median V_98%_ increased from 96.5% [IQR, 81.1%, 98.4%] to 98.1% [96.7%, 99.7%], and the median D_98%_ increased from 60.5 Gy(RBE) [53.1, 62.4] to 62.7 Gy(RBE) [62.2, 63.4] (*p* < 0.001, Figure 2a,b). Furthermore, the HI and CI of the dose distribution were significantly enhanced (median HI, as-scheduled plan vs. adaptive replan = 1.04 [1.02, 1.15] vs. 1.02 [1.02, 1.03], *p* < 0.001; CI, 0.61 [0.52–0.70] vs. 0.80 [0.69–0.84], *p* < 0.001) (Figure 2c).

**Figure 3:**
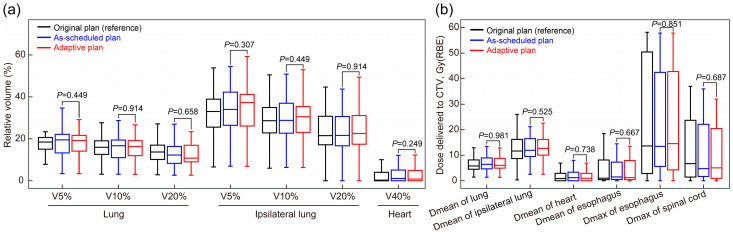
Comparison of normal tissue radiation exposure between the as-scheduled plan and adaptive replan in the (**a**) lung, ipsilateral lung, and heart and (**b**) esophagus and spinal cord. *p*-values were calculated using the paired Mann–Whitney U test. CTV, clinical target volume; RBE, relative biological effectiveness; V_90%_, V_95%_, and V_98%_, volume percentage of the clinical target volume covered by 90%, 95%, and 98% of the prescription dose, respectively.

**Figure A2:**
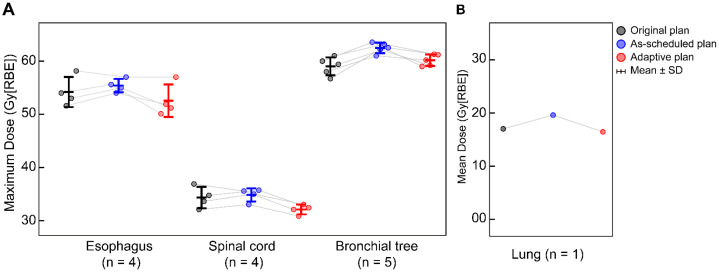
Cumulative dose variations in cases where adaptive radiotherapy was triggered due to excessive dose to (**A**) the esophagus (n = 4), spinal cord (n = 4), bronchial tree (n = 5), and (**B**) lung (n = 1) across the original plan, as-scheduled plan, and adaptive replan.

The plans of non-ART patients were separately compared with the as-scheduled plan and adaptive replan of the ART patients (Table A2). The results showed that the CTV coverage in plans of non-ART patients was significantly better than that in the as-scheduled plan of ART patients but comparable to their adaptive replan (*p* > 0.05). In brief, without adaptive replanning, these 27 patients would have had inferior target coverage compared with the general patients (non-ART patients), and a dose comparable to that of non-ART patients could be delivered with the adaptive replan. No significant differences in normal tissue coverage were observed between the adaptive and as-scheduled plans (all *p* > 0.05, Figure 3). Additionally, among patients who received ART due to an excessive normal tissue dose (n = 14), adaptive replanning effectively reduced the dose coverage to the affected regions (Figure A2).

### 3.4. Survival Outcomes and Toxicity Assessment

**Figure 4:**
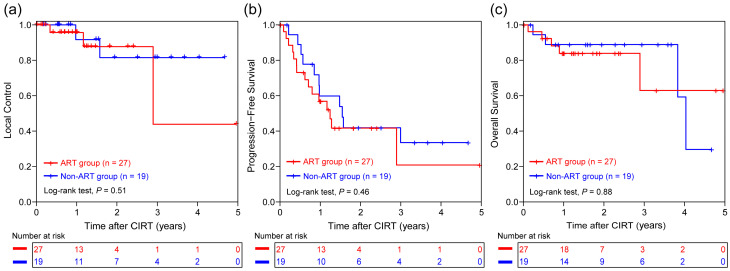
Kaplan–Meier curves for comparison of (**a**) local control, (**b**) progression-free survival, and (**c**) overall survival between the ART (n = 27) and non-ART (n = 19) groups. ART, adaptive radiotherapy; CIRT, carbon ion radiotherapy.

The median survival time was 48.4 months, with a median follow-up duration of 18.9 months (IQR: 10.9, 29.8 months). The LC, PFS, or OS was comparable between the ART and non-ART groups (all *p* > 0.05, Figure 4a–c), suggesting that ART enables patients with anatomical changes to achieve comparable outcomes to those with stable anatomy. No grade 3 or higher adverse events were observed in either group. The number of grade 2 adverse events was similar in the ART and non-ART groups; however, the incidence was lower in the ART group than in the non-ART group (Table 2). No chemotherapy-related serious adverse events were reported during treatment.

## 4. Discussion

This study evaluated the dosimetry benefits of ART in CIRT for LA-NSCLC in terms of accumulated dose. Adaptive replanning significantly improved target dose coverage, with acceptable normal tissue exposure and toxicity. These findings underscore the importance of ART for optimizing treatment precision and ensuring consistent therapeutic outcomes of CIRT for LA-NSCLC.

The Bragg peak and high RBE in CIRT indicate that even minor positional changes can significantly compromise the target dose distribution and increase radiation exposure to normal tissues. Therefore, timely adaptive adjustments to treatment plans are crucial for CIRT. Although the efficacy of ART in photon RT, particularly in addressing anatomical changes during treatment, is well-established [9,10,11,12,13,24], the results of its application in CIRT remain limited. In photon RT, ART is more commonly triggered by tumor shrinkage or anatomical changes [13,25]. In contrast, a suboptimal dose distribution or an increase in tumor volume was the primary trigger for adaptive replanning (57.9%, n = 22) in CIRT, rather than tumor shrinkage (7.9%, n = 3). This may be attributed to the shorter treatment duration of CIRT for LA-NSCLC at our center (4 weeks) compared with the typical 6- to 7-week courses of photon RT [12,13,25]. Larger tumors in CIRT can lead to greater mismatches between the dose distribution and tumor positioning, even with slight movements. Consequently, we observed that patients requiring adaptive replanning often had larger initial tumor volumes, which is consistent with the results of previous studies [7]. Therefore, owing to the physical and biological properties of CIRT, lung anatomy, and the heterogeneity of LA-NSCLC, ART is vital in clinical practice.

In the intragroup comparison, adaptive replanning significantly improved dosimetry coverage with acceptable normal tissue exposure compared with the as-scheduled plan in the ART group. The observed 2.2 Gy(RBE) increase in D_98%_ and 0.19 improvement in CI suggest that ART effectively compensates for interfractional anatomical variations, ensuring optimal dose delivery to the target. Cases where ART was triggered due to excessive exposure of normal tissue experienced a reduction in dose coverage after adaptive adjustment (Figure A2). These findings align with previous results [7], reinforcing the importance of ART in treatment planning optimization. However, a major concern regarding ART is the inability to determine, through intergroup comparison, whether the use of adaptive replanning or not influences clinical outcomes [26]. The clinical benefit of improved dose coverage should be continuously observed in future studies, including the long-term effects of ART on survival and tumor control in CIRT.

For the intergroup comparison, when comparing the as-scheduled plan for ART patients with non-ART patients, all dose parameters were inferior, but adaptive replanning restored the dose parameters to levels comparable to those of non-ART patients. This indirectly highlights the ability of ART to adaptively correct suboptimal plans and the necessity of triggering the plan change. Additionally, patients who triggered ART had comparable survival and toxicity outcomes to non-ART patients, demonstrating that ART enables patients with anatomical changes to maintain a comparable safety profile to those with stable anatomy. These findings suggest a possible role of ART in maintaining treatment quality in CIRT, especially in scenarios where tumor characteristics or anatomical changes challenge the initial treatment plans [26]. Notably, these survival data represent only a preliminary exploration, intended to demonstrate that implementing ART does not adversely affect survival [26]. Specifically, the non-ART group in this cohort represented patients who had been confirmed as not requiring adaptive replanning. Because it is difficult to recruit patients who appear to require ART for a prospective randomized trial comparing its administration versus omission, the potential survival benefit of ART could not be examined in detail. In addition, the small sample size and baseline imbalance (e.g., larger tumor volumes in the ART group) warrant confirmation of the outcomes through larger cohorts with more detailed subgroup analyses in the future.

Image acquisition and registration in CIRT is essential for the implementation of ART [10,11]. In the absence of fully developed online ART, weekly CT allows for the timely detection of tumor response, anatomical changes, and deterioration of dose distribution, enabling physicians to make necessary adjustments [7,10,11]. Given the relatively limited clinical evidence of ART for CIRT, using significantly few simulation CTs may result in inadequate monitoring, whereas excessive is time-consuming, increases clinical workload and additional irradiation exposure, and reduces patient throughput, affecting overall efficiency and even patient prognosis. A proton-RT study evaluated dose distribution using CT scans starting from the third or fourth week of treatment; however, their total fractions was 37, five per week [26]. In a previous study, we demonstrated the potential benefit of daily CT in patients with stage I NSCLC receiving 60 Gy(RBE) in 4 fractions [17]. Based on current findings, a weekly assessment frequency appears to provide a reasonable compromise for LA-NSCLC. We found that the median fraction triggering ART was 7 (second week), but there were also cases where ART was indicated at the beginning or near the end of CIRT. Notably, the timing of the decision to trigger ART and its actual implementation differ. Offline replanning requires dose recalculations and approval processes, inevitably causing delays. In clinical practice, as CIRT delivers only one irradiation field per day, priority is given to unmodified fields, while modified fields are implemented once replanning is complete to ensure uninterrupted treatment and prevent delays. Meanwhile, this result was consistent with another CIRT study, which reported a total of 16–23 fractions for CIRT, with ART triggered at a median of 8 fractions (range, 2–18) [7]. Therefore, for CIRT in LA-NSCLC, weekly CT simulation scans may be the minimum requirement. However, owing to the small sample sizes, the optimal ART strategy for CIRT still requires comprehensive evaluation. Another noteworthy point is that, in passive scattering-based offline ART, patient-specific compensators often require adjustment to implement adaptive plans [18], which may limit flexibility. In contrast, active scanning (spot scanning) delivers dose layer-by-layer to the target volume, enabling faster plan adaptation and potentially improving workflow efficiency [27,28]. Nevertheless, timely evaluation of dose distribution remains essential for both techniques. Although weekly CT scans have inherent limitations for fully implementing ART, they represent a practical compromise in the current setting where online ART technology is not yet fully established. Furthermore, to calculate the cumulative dose, it is necessary to employ DIR for matching the correspondence between each voxel in two CT image sets [29]. This process involves acquiring weekly CT images, integrating the weekly dose distribution onto these images, and summing them to obtain the total cumulative dose distribution. Prior studies have confirmed the accuracy of the DIR technique in calculating the cumulative dose distribution in CIRT [17,29]. Additionally, offline ART is a time-consuming process. DIR can reduce contouring and planning time [11,25], thereby alleviating the workload associated with offline ART.

The current study is limited first by its retrospective nature and small sample size, which may reduce statistical power and increase the risk of both Type I and Type II errors; therefore, the findings should be considered preliminary results of ART in CIRT, requiring further prospective validation. Second, for ART prediction, who should undergo ART and when it should be implemented were not clarified. This is a key area for future studies. Third, the lymph node dose coverage was not evaluated. No cases in this cohort underwent ART due to inadequate nodal dose coverage, as the lymph nodes were generally well aligned with the bony structures, whereas the primary tumor was more strongly influenced by other factors such as respiratory motion. At our institute, bone matching was adopted for every treatment session; therefore, most lymph nodes in this study were irradiated accurately. In addition, approximately half of the patients (43.5%) had no lymph node metastases, which further limited the analysis and could introduce substantial bias. Future prospective studies are warranted to evaluate the dosimetric and clinical impact of ART on nodal regions, particularly in patients with bulky nodal disease or complex anatomical changes. Fourth, the current use of offline ART significantly increases the workload. In addition, this study did not include a detailed quantitative evaluation of how the mitigation strategy for offline ART delays influenced clinical outcomes, workflow efficiency, and dose delivery uniformity. Due to the retrospective nature of this study and the lack of systematically collected workflow data, there are limitations in performing such analyses; future prospective studies incorporating structured workflow metrics are warranted to address this issue. Finally, while online ART remains technically challenging in CIRT, it represents a promising direction for streamlining adaptation and improving real-time precision, and advances in automated prediction models and contouring, planning, and quality assurance software may alleviate the current workflow burden [30].

## 5. Conclusions

ART in CIRT improves target coverage without increasing normal tissue exposure or toxicity. Weekly CT-based assessment facilitates timely cumulative dose assessment of the target. These findings provide a foundation for future prospective trials evaluating the clinical benefits of ART in CIRT.

## Figures and Tables

**Table 1:** Patient characteristics.

Characteristic	Overall	Non-ART	ART	*p* Value
	n = 46	n = 19	n = 27	
Age, mean (SD)	73.6 (9.5)	75.6 (7.9)	72.3 (10.5)	0.304
Sex (%)				0.877
Male	38 (82.6)	15 (78.9)	23 (85.2)	
Female	8 (17.4)	4 (21.1)	4 (14.8)	
Height (cm), mean (SD)	163.6 (7.4)	161.8 (8.2)	164.9 (6.6)	0.166
Weight (kg), mean (SD)	57.6 (10.7)	55.6 (11.8)	59.0 (9.8)	0.237
BMI, mean (SD)	21.4 (3.1)	21.1 (3.3)	21.7 (3.0)	0.503
Past Medical History (%)				1.000
Lung-related diseases	23 (50.0)	9 (47.4)	14 (51.8)	
Other	23 (50.0)	10 (52.6)	13 (48.2)	
Tumor Site (%)				0.833
Right	27 (58.7)	12 (63.2)	15 (55.6)	
Left	19 (41.3)	7 (36.8)	12 (44.4)	
Tumor Location (%)				0.118
Central type	17 (37.0)	4 (21.1)	13 (48.1)	
Peripheral type	29 (63.0)	15 (78.9)	14 (51.9)	
Pulmonary Lobe (%)				0.288
Upper	20 (43.5)	6 (31.6)	14 (51.8)	
Lower	26 (56.5)	13 (68.4)	13 (48.2)	
Physiology (%)				0.405
SCC	27 (58.7)	9 (47.4)	18 (66.7)	
Adenocarcinoma	7 (15.2)	4 (21.0)	3 (11.1)	
Clinical diagnosis (histology unknown)	12 (26.1)	6 (31.6)	6 (22.2)	
Maximum tumor size (mm), mean (SD)	43.51 (19.7)	38.1 (19.3)	47.3 (19.5)	0.078
Tumor volume (ml), median [IQR]	36.1 [18.9,66.7]	25.6 [2.7,45.2]	42.6 [21.2,90.4]	0.037
N Stage (%)				0.580
N0	20 (43.5)	7 (36.8)	13 (48.1)	
N+	26 (56.5)	12 (63.2)	14 (51.9)	
TNM Stage (%)				0.486
IIB	24 (52.2)	11 (57.9)	13 (48.2)	
IIIA	11 (23.9)	5 (26.3)	6 (22.2)	
IIIB	8 (17.4)	3 (15.8)	5 (18.5)	
IIIC	3 (6.5)	0 (0.00)	3 (11.1)	
Smoking (%)				0.178
No	9 (19.6)	6 (31.6)	3 (11.1)	
Yes	37 (80.4)	13 (68.4)	24 (88.9)	
Drinking (%)				0.846
No	31 (67.4)	12 (63.2)	19 (70.4)	
Yes	15 (32.6)	7 (36.8)	8 (29.6)	
Chemotherapy (%)				0.213
No	39 (84.8)	18 (94.7)	21 (77.8)	
Yes	7 (15.2)	1 (5.3)	6 (22.2)	
Follow-up time (months), median [IQR]	18.9 [10.9, 29.8]	23.3 [12.1, 41.6]	15.4 [11.1, 25.0]	0.190

Abbreviations: ART, adaptive radiotherapy; BMI, body mass index; SD, standard deviation; IQR, interquartile range; SCC, squamous cell carcinoma; TNM, tumor-node-metastasis staging according to the American Joint Committee on Cancer, 8th edition.

**Table 2:** Grade 2 adverse events related, or possibly related, to CIRT.

Type of Adverse Effect	ART Group, n = 27 (%)	Non-ART Group, n = 19 (%)
Acute Reactions	7 (25.9)	5 (26.3)
Dermatitis	1 (3.7)	1 (5.3)
Pneumonitis	1 (3.7)	1 (5.3)
Esophagitis	3 (11.1)	2 (10.5)
Chest wall pain	0 (0)	1 (5.3)
Leukopenia	1 (3.7)	0 (0)
Anemia	0 (0)	0 (0)
Thrombocytopenia	1 (3.7)	0 (0)
Late Reactions	2 (7.4)	4 (21.1)
Dermatitis	0 (0)	0 (0)
Pneumonitis	0 (0)	2 (10.5)
Esophagitis	2 (7.4)	1 (5.3)
Chest wall	0 (0)	0 (0)
Rib fractures	0 (0)	1 (5.3)

## Data Availability

The raw data supporting the conclusions of this article will be made available by the authors on request.

## Abbreviations

The following abbreviations are used in this manuscript:

CIRT	Carbon ion radiotherapy
LA-NSCLC	Locally advanced non-small cell lung cancer
RBE	Relative biological effectiveness
CT	Computed tomography
ART	Adaptive radiotherapy
DIR	Deformable image registration
BMI	Body mass index
SD	Standard deviation
IQR	Interquartile range
SCC	Squamous cell carcinoma
TNM	Tumor–node–metastasis
GTV	Gross tumor volume
CTV	Clinical target volume
IM	Internal margin
HI	Homogeneity index
CI	Conformity index
LC	Local control
PFS	Progression-free survival
OS	Overall survival

## Appendix A

**Table A1:** Dose–volume constraints for organs at risk.

Organs at Risk	Dose Limits
Lungs normal ^a^	V_30 Gy(RBE)_ < 15%, Minimizing lung dose as much as possible
Esophagus	Maximum < 60 Gy(RBE) (Dmax of ≤56 Gy is preferable)
Spinal cord	Maximum dose < 50 Gy(RBE)
	V_30 Gy(RBE)_ < 0.7 cc
Trachea	Maximum dose < 60 Gy(RBE) (Dmax of ≤56 Gy is preferable)
Bronchus	Maximum dose < 60 Gy(RBE) (Dmax of ≤56 Gy is preferable)

^a^ Lungs normal contour is the whole lung with the target area (GTV) removed. Abbreviations: RBE, relative biological effectiveness; cc, cubic centimeter; Dmax, maximum dose.

**Table A2:** Comparison of CTV coverage between ART and non-ART patients.

Parameter	Non-ART	ART		*p* Value	*p* Value
	As-Scheduled Plan (n = 19)	As-Scheduled Plan (n = 27)	Adaptive Plan (n = 27)	Non-ART vs. As-Scheduled Plan	Non-ART vs. Adaptive Plan
V_90%_ (%)	100.00 [99.96, 100.00]	99.55 [94.97, 99.97]	99.99 [99.84, 100.00]	<0.001	0.780
V_95%_ (%)	100.00 [99.64, 100.00]	98.65 [86.30, 99.80]	99.76 [99.19, 100.00]	<0.001	0.772
V_98%_ (%)	99.59 [95.70, 99.99]	96.48 [81.10, 98.35]	98.10 [96.68, 99.73]	0.004	0.260
D_90%_ [Gy(RBE)]	63.91 [63.23, 63.98]	63.12 [59.14, 63.84]	63.78 [63.58, 64.02]	0.032	0.156
D_95%_ [Gy(RBE)]	63.50 [62.86, 63.89]	62.07 [56.22, 63.38]	63.50 [63.05, 63.86]	0.006	0.075
D_98%_ [Gy(RBE)]	63.35 [62.42, 63.78]	60.47 [53.12, 62.40]	62.73 [62.21, 63.39]	<0.001	0.448
HI	1.02 [1.02, 1.03]	1.04 [1.02, 1.15]	1.02 [1.02, 1.03]	<0.001	0.632
CI	0.74 [0.61, 0.86]	0.61 [0.52, 0.70]	0.80 [0.69, 0.84]	0.020	0.511

*p*-values were calculated using the Mann–Whitney U test. Abbreviations: CI, conformal index; HI, homogeneity index; RBE, relative biological effectiveness.
